# 5-[(4-Meth­oxy­benz­yl)sulfan­yl]-2-methyl-1,3,4-thia­diazole

**DOI:** 10.1107/S1600536810051858

**Published:** 2010-12-18

**Authors:** Hoong-Kun Fun, Suchada Chantrapromma, B. Chandrakantha, Arun M. Isloor, Prakash Shetty

**Affiliations:** a X-ray Crystallography Unit, School of Physics, Universiti Sains Malaysia, 11800 USM, Penang, Malaysia; b Crystal Materials Research Unit, Department of Chemistry, Faculty of Science, Prince of Songkla University, Hat-Yai, Songkhla 90112, Thailand; c Department of Chemistry, Manipal Institute of Technology, Manipal 576 104, India; d Organic Chemistry Division, Department of Chemistry, National Institute of Technology-Karnataka, Surathkal, Mangalore 575 025, India; e Department of Printing, Manipal Institute of Technology, Manipal 576 104, India

## Abstract

The title mol­ecule, C_11_H_12_N_2_OS_2_, is twisted with a dihedral angle of 83.63 (12)° between the 1,3,4-thia­diazole and benzene rings. The meth­oxy group deviates slightly from the attached benzene ring, with a C—C—O—C torsion angle of 4.2 (4)°. In the crystal, mol­ecules are linked by weak C—H⋯N inter­actions and stacked along the *c* axis.

## Related literature

For bond-length data, see: Allen *et al.* (1987 ▶). For a related structure, see: Wang *et al.* (2010 ▶). For background to and applications of thia­diazole derivatives, see: Bernard *et al.* (1985 ▶); Chandrakantha *et al.* (2010 ▶); El-Sabbagh *et al.* (2009) ▶; Isloor *et al.* (2010 ▶); Kalluraya *et al.* (2004 ▶). For the stability of the temperature controller, see: Cosier & Glazer (1986 ▶).
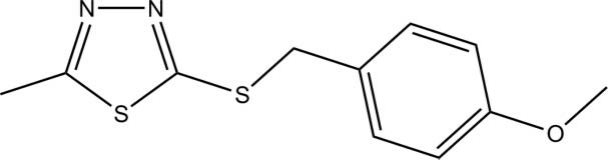


## Experimental

### 

#### Crystal data

C_11_H_12_N_2_OS_2_
*M* *_r_* = 252.35
Monoclinic, 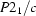

*a* = 14.7765 (4) Å
*b* = 8.6916 (3) Å
*c* = 9.7339 (3) Å
β = 96.477 (1)°
*V* = 1242.16 (7) Å^3^
*Z* = 4
Mo *K*α radiation
μ = 0.41 mm^−1^
*T* = 296 K
0.25 × 0.19 × 0.03 mm

#### Data collection

Bruker APEXII CCD area-detector diffractometer
Absorption correction: multi-scan (*SADABS*; Bruker, 2005 ▶) *T*_min_ = 0.907, *T*_max_ = 0.987
11429 measured reflections
2828 independent reflections
1660 reflections with *I* > 2σ(*I*)
*R*_int_ = 0.040

#### Refinement

*R*[*F* ^2^ > 2σ(*F* ^2^)] = 0.052
*wR*(*F* ^2^) = 0.118
*S* = 1.02
2828 reflections
147 parameters
H-atom parameters constrained
Δρ_max_ = 0.23 e Å^−3^
Δρ_min_ = −0.19 e Å^−3^

### 

Data collection: *APEX2* (Bruker, 2005 ▶); cell refinement: *SAINT* (Bruker, 2005 ▶); data reduction: *SAINT*; program(s) used to solve structure: *SHELXTL* (Sheldrick, 2008 ▶); program(s) used to refine structure: *SHELXTL*; molecular graphics: *SHELXTL*; software used to prepare material for publication: *SHELXTL* and *PLATON* (Spek, 2009 ▶).

## Supplementary Material

Crystal structure: contains datablocks global, I. DOI: 10.1107/S1600536810051858/is2640sup1.cif

Structure factors: contains datablocks I. DOI: 10.1107/S1600536810051858/is2640Isup2.hkl

Additional supplementary materials:  crystallographic information; 3D view; checkCIF report

## Figures and Tables

**Table 1:** Hydrogen-bond geometry (Å, °)

*D*—H⋯*A*	*D*—H	H⋯*A*	*D*⋯*A*	*D*—H⋯*A*
C1—H1*B*⋯N1^i^	0.96	2.59	3.532 (4)	164

Symmetry code: (i) 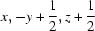.

**Fig. 1.**
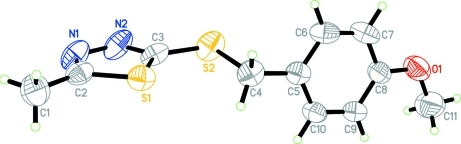
The molecular structure of the title compound, showing 40% probability displacement ellipsoids and the atom-numbering scheme.

**Fig. 2.**
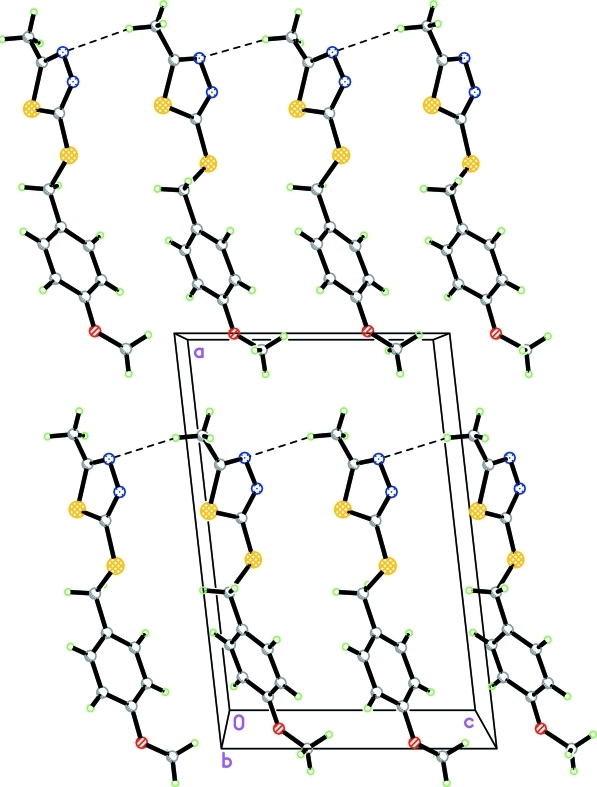
The crystal packing of the title compound viewed along the b axis. C—H···N weak interactions are shown as dashed lines.

## supplementary crystallographic information

### Comment

Thiadiazole are a class of heterocyclic compounds having a five membered ring. They occur in nature and are predominant among all types of pharmaceuticals, agrochemicals and veterinary products (El-Sabbagh *et al.*, 2009). The amino and mercapto groups in thiadiazole are readily-accessible nucleophilic centers. 1,3,4-Thiadiazole exhibit a wide spectrum of biological activities (Bernard *et al.*, 1985). Due to the presence of the –N—C—S moiety (Kalluraya *et al.*, 2004), they are found to be used as antibacterial, antimicrobial and anti-inflammatory agents (Chandrakantha *et al.*, 2010). Antibacterial and antifungal (Isloor *et al.*, 2010) activities of the azoles are most widely studied and azoles are also used as antimicrobial agents. Herein we report the crystal structure of the title 1,3,4-thiadiazole derivative, (I).

The molecule of (I) (Fig. 1) is twisted with a dihedral angle between the 1,3,4-thiadiazole and benzene rings being 83.63 (12)°. Atoms C3, S2, C4 and C5 lie nearly on the same plane with *r.m.s*. 0.0517 (5) Å and the torsion angle C3–S2–C4–C5 = 172.25 (18)°. The mean plane through C3/S2/C4/C5 makes the dihedral angles of 9.02 (15) and 75.92 (16)° with the 1,3,4-thiadiazole and benzene rings, respectively. The methoxy group is slightly deviated with respect to the attached benzene ring with the torsion angle C11–O1–C8–C9 = 4.2 (4)°. The bond distances are of normal values (Allen *et al.*, 1987) and are comparable with the related structure (Wang *et al.*, 2010).

In the crystal packing (Fig. 2), the molecules are linked by C1—H1B···N1 weak interactions (Table 1) and stacked along the *c* axis. S···N [3.340 (2) Å] short contacts (symmetry codes: *x*, 1/2 - *y*, 1/2 + *z* and *x*, 1/2 - *y*, -1/2 + *z*) are presented in the crystal.

### Experimental

The title compound was synthesized by adding 4-methoxybenzylbromide (3.02 g, 0.0151 mol) dropwise to a stirred solution of 5-methyl-1,3,4-thiadiazole-2-thiol (2.00 g, 0.0151 mol) and anhydrous potassiumcarbonate (4.16 g, 0.03 mol) in dry acetonitrile (50 ml) at room temperature and the reaction mixture was stirred at room temperature for 5 h. After the completion of reaction, the reaction mixture was filtered and the filtrate was concentrated. The crude product was recrystallized with hot ethanol to afford the title compound as yellow solid (2.00 g, yield 57%). Yellow plate-shaped single crystals of the title compound suitable for *x*-ray structure determination were recrystalized from ethanol by the slow evaporation of the solvent at room temperature after several days (m.p. 413–415 K).

### Refinement

All H atoms were positioned geometrically and allowed to ride on their parent atoms, with *d*(C—H) = 0.93 Å for aromatic, 0.97 Å for CH_2_ and 0.96 Å for CH_3_ atoms. The *U*_iso_(H) values were constrained to be 1.5*U*_eq_(C) for methyl H atoms and 1.2*U*_eq_(C) for the remaining H atoms. A rotating group model was used for the methyl groups.

### Crystal data

**Table:**

C_11_H_12_N_2_OS_2_	*F*(000) = 528
*M**_r_* = 252.35	*D*_x_ = 1.349 Mg m^−^^3^
Monoclinic, *P*2_1_/*c*	Melting point = 413–415 K
Hall symbol: -P 2ybc	Mo *K*α radiation, λ = 0.71073 Å
*a* = 14.7765 (4) Å	Cell parameters from 2828 reflections
*b* = 8.6916 (3) Å	θ = 2.7–27.5°
*c* = 9.7339 (3) Å	µ = 0.41 mm^−^^1^
β = 96.477 (1)°	*T* = 296 K
*V* = 1242.16 (7) Å^3^	Plate, yellow
*Z* = 4	0.25 × 0.19 × 0.03 mm

### Data collection

**Table:**

Bruker APEXII CCD area-detector diffractometer	2828 independent reflections
Radiation source: sealed tube	1660 reflections with *I* > 2σ(*I*)
graphite	*R*_int_ = 0.040
φ and ω scans	θ_max_ = 27.5°, θ_min_ = 2.7°
Absorption correction: multi-scan (*SADABS*; Bruker, 2005)	*h* = −19→19
*T*_min_ = 0.907, *T*_max_ = 0.987	*k* = −11→11
11429 measured reflections	*l* = −12→12

### Refinement

**Table:**

Refinement on *F*^2^	Primary atom site location: structure-invariant direct methods
Least-squares matrix: full	Secondary atom site location: difference Fourier map
*R*[*F*^2^ > 2σ(*F*^2^)] = 0.052	Hydrogen site location: inferred from neighbouring sites
*wR*(*F*^2^) = 0.118	H-atom parameters constrained
*S* = 1.02	*w* = 1/[σ^2^(*F*_o_^2^) + (0.0454*P*)^2^ + 0.2899*P*] where *P* = (*F*_o_^2^ + 2*F*_c_^2^)/3
2828 reflections	(Δ/σ)_max_ = 0.001
147 parameters	Δρ_max_ = 0.23 e Å^−^^3^
0 restraints	Δρ_min_ = −0.19 e Å^−^^3^

### Special details

**Table:**

Geometry. All e.s.d.'s (except the e.s.d. in the dihedral angle between two l.s. planes) are estimated using the full covariance matrix. The cell e.s.d.'s are taken into account individually in the estimation of e.s.d.'s in distances, angles and torsion angles; correlations between e.s.d.'s in cell parameters are only used when they are defined by crystal symmetry. An approximate (isotropic) treatment of cell e.s.d.'s is used for estimating e.s.d.'s involving l.s. planes.
Refinement. Refinement of *F*^2^ against ALL reflections. The weighted *R*-factor *wR* and goodness of fit *S* are based on *F*^2^, conventional *R*-factors *R* are based on *F*, with *F* set to zero for negative *F*^2^. The threshold expression of *F*^2^ > σ(*F*^2^) is used only for calculating *R*-factors(gt) *etc*. and is not relevant to the choice of reflections for refinement. *R*-factors based on *F*^2^ are statistically about twice as large as those based on *F*, and *R*- factors based on ALL data will be even larger.

### Fractional atomic coordinates and isotropic or equivalent isotropic displacement parameters (Å^2^)

**Table:**

	*x*	*y*	*z*	*U*_iso_*/*U*_eq_	
S1	0.43447 (5)	0.19481 (8)	0.95596 (7)	0.0631 (2)	
S2	0.56474 (5)	0.40517 (9)	0.81756 (8)	0.0805 (3)	
O1	0.99330 (14)	0.4254 (2)	0.7932 (2)	0.0899 (7)	
N1	0.30610 (16)	0.3239 (3)	0.8041 (2)	0.0770 (7)	
N2	0.38487 (18)	0.3952 (3)	0.7726 (2)	0.0795 (7)	
C1	0.24763 (19)	0.1218 (4)	0.9455 (3)	0.0849 (9)	
H1A	0.1893	0.1624	0.9098	0.127*	
H1B	0.2534	0.1234	1.0447	0.127*	
H1C	0.2529	0.0178	0.9141	0.127*	
C2	0.32087 (18)	0.2174 (3)	0.8958 (2)	0.0616 (7)	
C3	0.45726 (18)	0.3383 (3)	0.8427 (2)	0.0609 (7)	
C4	0.63995 (18)	0.2718 (3)	0.9180 (3)	0.0672 (7)	
H4A	0.6353	0.2848	1.0159	0.081*	
H4B	0.6239	0.1665	0.8925	0.081*	
C5	0.73488 (17)	0.3071 (3)	0.8867 (2)	0.0581 (7)	
C6	0.7820 (2)	0.4335 (3)	0.9444 (3)	0.0697 (8)	
H6A	0.7551	0.4956	1.0062	0.084*	
C7	0.8673 (2)	0.4688 (3)	0.9122 (3)	0.0738 (8)	
H7A	0.8977	0.5539	0.9528	0.089*	
C8	0.90874 (18)	0.3794 (3)	0.8200 (3)	0.0624 (7)	
C9	0.86360 (19)	0.2522 (3)	0.7635 (3)	0.0678 (7)	
H9A	0.8909	0.1892	0.7029	0.081*	
C10	0.77752 (18)	0.2183 (3)	0.7970 (3)	0.0653 (7)	
H10A	0.7475	0.1324	0.7573	0.078*	
C11	1.0358 (2)	0.3427 (5)	0.6936 (4)	0.1167 (14)	
H11A	1.0964	0.3813	0.6905	0.175*	
H11B	1.0014	0.3548	0.6046	0.175*	
H11C	1.0385	0.2356	0.7180	0.175*	

### Atomic displacement parameters (Å^2^)

**Table:**

	*U*^11^	*U*^22^	*U*^33^	*U*^12^	*U*^13^	*U*^23^
S1	0.0756 (5)	0.0557 (4)	0.0557 (4)	0.0085 (3)	−0.0024 (3)	0.0090 (3)
S2	0.0880 (6)	0.0761 (6)	0.0793 (5)	0.0158 (4)	0.0181 (4)	0.0313 (4)
O1	0.0779 (14)	0.0861 (16)	0.1076 (16)	−0.0229 (11)	0.0186 (12)	−0.0254 (12)
N1	0.0787 (17)	0.0942 (19)	0.0594 (14)	0.0339 (14)	0.0139 (12)	0.0159 (14)
N2	0.0849 (17)	0.0896 (18)	0.0665 (15)	0.0385 (15)	0.0200 (13)	0.0277 (14)
C1	0.079 (2)	0.094 (2)	0.079 (2)	−0.0025 (18)	−0.0034 (16)	0.0020 (18)
C2	0.0744 (18)	0.0651 (18)	0.0449 (14)	0.0152 (14)	0.0056 (13)	−0.0067 (13)
C3	0.0806 (18)	0.0570 (17)	0.0466 (14)	0.0229 (14)	0.0137 (13)	0.0045 (12)
C4	0.0779 (19)	0.0640 (18)	0.0595 (16)	0.0065 (14)	0.0061 (14)	0.0151 (14)
C5	0.0708 (17)	0.0520 (16)	0.0507 (14)	0.0007 (13)	0.0035 (13)	0.0073 (13)
C6	0.099 (2)	0.0566 (18)	0.0563 (16)	−0.0032 (16)	0.0208 (15)	−0.0093 (14)
C7	0.099 (2)	0.0593 (18)	0.0631 (17)	−0.0209 (16)	0.0091 (16)	−0.0141 (15)
C8	0.0659 (17)	0.0571 (17)	0.0626 (17)	−0.0049 (14)	0.0002 (13)	−0.0044 (14)
C9	0.0701 (18)	0.0569 (17)	0.0758 (18)	0.0007 (14)	0.0062 (14)	−0.0168 (15)
C10	0.0701 (18)	0.0512 (17)	0.0726 (18)	−0.0057 (13)	−0.0011 (14)	−0.0122 (14)
C11	0.092 (2)	0.105 (3)	0.162 (4)	−0.014 (2)	0.051 (3)	−0.037 (3)

### Geometric parameters (Å, °)

**Table:**

S1—C3	1.723 (3)	C4—H4B	0.9700
S1—C2	1.725 (3)	C5—C10	1.371 (3)
S2—C3	1.734 (3)	C5—C6	1.386 (4)
S2—C4	1.814 (3)	C6—C7	1.367 (4)
O1—C8	1.365 (3)	C6—H6A	0.9300
O1—C11	1.410 (3)	C7—C8	1.382 (4)
N1—C2	1.288 (3)	C7—H7A	0.9300
N1—N2	1.383 (3)	C8—C9	1.373 (4)
N2—C3	1.300 (3)	C9—C10	1.380 (3)
C1—C2	1.488 (4)	C9—H9A	0.9300
C1—H1A	0.9600	C10—H10A	0.9300
C1—H1B	0.9600	C11—H11A	0.9600
C1—H1C	0.9600	C11—H11B	0.9600
C4—C5	1.500 (3)	C11—H11C	0.9600
C4—H4A	0.9700		
			
C3—S1—C2	87.33 (13)	C10—C5—C4	121.5 (2)
C3—S2—C4	102.99 (12)	C6—C5—C4	121.2 (2)
C8—O1—C11	118.1 (2)	C7—C6—C5	121.3 (2)
C2—N1—N2	113.2 (2)	C7—C6—H6A	119.4
C3—N2—N1	112.1 (2)	C5—C6—H6A	119.4
C2—C1—H1A	109.5	C6—C7—C8	120.7 (3)
C2—C1—H1B	109.5	C6—C7—H7A	119.7
H1A—C1—H1B	109.5	C8—C7—H7A	119.7
C2—C1—H1C	109.5	O1—C8—C9	125.0 (2)
H1A—C1—H1C	109.5	O1—C8—C7	116.2 (2)
H1B—C1—H1C	109.5	C9—C8—C7	118.8 (3)
N1—C2—C1	123.7 (3)	C8—C9—C10	119.8 (3)
N1—C2—S1	113.5 (2)	C8—C9—H9A	120.1
C1—C2—S1	122.8 (2)	C10—C9—H9A	120.1
N2—C3—S1	113.7 (2)	C5—C10—C9	122.2 (2)
N2—C3—S2	120.7 (2)	C5—C10—H10A	118.9
S1—C3—S2	125.53 (16)	C9—C10—H10A	118.9
C5—C4—S2	106.83 (17)	O1—C11—H11A	109.5
C5—C4—H4A	110.4	O1—C11—H11B	109.5
S2—C4—H4A	110.4	H11A—C11—H11B	109.5
C5—C4—H4B	110.4	O1—C11—H11C	109.5
S2—C4—H4B	110.4	H11A—C11—H11C	109.5
H4A—C4—H4B	108.6	H11B—C11—H11C	109.5
C10—C5—C6	117.3 (2)		
			
C2—N1—N2—C3	0.2 (3)	S2—C4—C5—C6	76.6 (3)
N2—N1—C2—C1	−178.9 (2)	C10—C5—C6—C7	0.5 (4)
N2—N1—C2—S1	0.9 (3)	C4—C5—C6—C7	−177.7 (2)
C3—S1—C2—N1	−1.3 (2)	C5—C6—C7—C8	0.4 (4)
C3—S1—C2—C1	178.5 (2)	C11—O1—C8—C9	4.2 (4)
N1—N2—C3—S1	−1.3 (3)	C11—O1—C8—C7	−176.3 (3)
N1—N2—C3—S2	178.12 (18)	C6—C7—C8—O1	179.1 (2)
C2—S1—C3—N2	1.5 (2)	C6—C7—C8—C9	−1.4 (4)
C2—S1—C3—S2	−177.88 (18)	O1—C8—C9—C10	−179.1 (3)
C4—S2—C3—N2	−171.8 (2)	C7—C8—C9—C10	1.5 (4)
C4—S2—C3—S1	7.5 (2)	C6—C5—C10—C9	−0.4 (4)
C3—S2—C4—C5	172.25 (18)	C4—C5—C10—C9	177.8 (2)
S2—C4—C5—C10	−101.5 (3)	C8—C9—C10—C5	−0.6 (4)

### Hydrogen-bond geometry (Å, °)

**Table:**

*D*—H···*A*	*D*—H	H···*A*	*D*···*A*	*D*—H···*A*
C1—H1B···N1^i^	0.96	2.59	3.532 (4)	164

Symmetry codes: (i) *x*, −*y*+1/2, *z*+1/2.
